# Inpatient burden of juvenile dermatomyositis among children in the United States

**DOI:** 10.1186/s12969-018-0286-1

**Published:** 2018-11-13

**Authors:** Michael C. Kwa, Jonathan I. Silverberg, Kaveh Ardalan

**Affiliations:** 1 Department of Dermatology, Northwestern University Feinberg School of Medicine, Chicago, IL 60611 USA; 2 Departments of Dermatology, Preventive Medicine and Medical Social Sciences, Northwestern University Feinberg School of Medicine, Chicago, IL 60611 USA; 3 Division of Rheumatology, Departments of Pediatrics and Medical Social Sciences, Ann & Robert H. Lurie Children’s Hospital of Chicago/Northwestern University Feinberg School of Medicine, 225 E Chicago Ave Box 50, Chicago, IL 60611 USA

**Keywords:** Juvenile dermatomyositis, Epidemiology, Cost of care

## Abstract

**Background:**

Juvenile dermatomyositis (JDM) is a rare autoimmune disease that causes significant morbidity and quality of life impairment. Little is known about the inpatient burden of JDM in the US. Our goal was to determine the prevalence and risk factors for hospitalization with juvenile dermatomyositis and assess inpatient burden of JDM.

**Methods:**

Data on 14,401,668 pediatric hospitalizations from the 2002–2012 Nationwide Inpatient Sample (NIS) was analyzed. ICD-9-CM coding was used to identify hospitalizations with a diagnosis of JDM.

**Results:**

There were 909 and 495 weighted admissions with a primary or secondary diagnosis of JDM, respectively. In multivariable logistic regression models with stepwise selection, female sex (logistic regression; adjusted odds ratio [95% confidence interval]) (2.22 [2.05–2.42]), non-winter season (fall: 1.18[1.06–1.33]; spring (1.13 [1.01–1.27]; summer (1.53 [1.37–1.71]), non-Medicaid administered government insurance coverage (2.59 [2.26–2.97]), and multiple chronic conditions (2–5: 1.41[1.30–1.54]; 6+: 1.24[1.00–1.52]) were all associated with higher rates of hospitalization for JDM. The weighted total length of stay (LOS) and inflation-adjusted cost of care for patients with a primary inpatient diagnosis of JDM was 19,159 days and $49,339,995 with geometric means [95% CI] of 2.50 [2.27–2.76] days and $7350 [$6228–$8674], respectively. Costs of hospitalization in primary JDM and length of stay and cost in secondary JDM were significantly higher compared to those without JDM. Notably, race/ethnicity was associated with increased LOS (log-linear regression; adjusted beta [95% confidence interval]) (Hispanic: 0.28 [0.14–0.41]; other non-white: 0.59 [0.31–0.86]) and cost of care (Hispanic: 0.30 [0.05–0.55]).

**Conclusion:**

JDM contributes to both increased length of hospitalization and inpatient cost of care. Non-Medicaid government insurance was associated with higher rates of hospitalization for JDM while Hispanic and other non-white racial/ethnic groups demonstrated increased LOS and cost of care.

## Background

Juvenile dermatomyositis (JDM) is a rare autoimmune disease characterized by proximal muscle weakness concurrent with specific cutaneous manifestations. [1] In the United States, JDM has an estimated incidence of 2–4 per million children per year. [2] While improved management of the disease has led to reduced mortality in recent years, up to 40% JDM patients continue to have active disease despite treatment. [3, 4] Ongoing disease activity, cumulative damage, and aggressive immunosuppressive treatments remain a concern for long term outcomes and quality of life. [4] Previous studies have compared cost of specific JDM treatment regimens [5], evaluated the inpatient burden of adult dermatomyositis [6], and examined economic burden of other childhood inflammatory conditions such as JIA [7]. However, little is known about the inpatient burden of JDM in the United States. As a result, use of a comprehensive, national inpatient database could help to elucidate the economic burden posed by JDM.

Previous studies found racial/ethnic and socioeconomic differences in hospitalization rates and outcomes for stroke [8], cardiovascular disease [9], asthma [10], acute respiratory illness [11], pemphigus [12], and adult dermatomyositis [6]. We hypothesized that JDM is also associated with similar racial/ethnic and socioeconomic differences, possibly related to lack of insurance coverage and reduced access to specialty care, such as rheumatology and dermatology. In the present study, we analyzed the prevalence and predictors of hospitalization, cost of care and length of stay in US patients with JDM.

## Methods

### Data source

The 2002–2012 Nationwide Inpatient Sample (NIS) provided by the Healthcare Cost and Utilization Project (HCUP) from the Agency for Healthcare Research and Quality (AHRQ) was analyzed. Each year of NIS contains an approximately 20% stratified representative sample of all inpatient hospitalizations in the United States. Sample weights were created by NIS that factored the sampling design of hospitals in the US. These sample weights are needed to provide representative estimates of hospital discharges across the whole country. All data were de-identified and no attempts were made to identify any of the individuals in the database. All parties with access to the HCUP were compliant to HCUP’s formal data use agreement. This study was deemed exempt by the institutional review board at Northwestern University.

### Identification of JDM

The databases were searched for a primary and/or secondary diagnosis of JDM using the International Classification of Diseases, Ninth Revision, Clinical Modification (*ICD-9-CM)* code 710.3. The primary diagnosis was defined in NIS as the condition chiefly responsible for admission to the hospital for care. A previous study validated the use of the discharge diagnosis code 710.3 in the inpatient setting for the study of dermatomyositis. [13] Patients with *ICD-9-CM* diagnostic codes of 701.0/710.1 (scleroderma), 710.0 (systemic lupus erythematosus), 710.4 (polymyositis), 710.8 (mixed connective tissue disease), and 710.9 (undifferentiated connective tissue disease) were excluded to minimize misclassification. The control group included all hospitalizations without any diagnosis of JDM, yielding a representative cohort of US pediatric hospitalizations.

### Data processing and statistics

All data analyses and statistical processes were performed using SAS version 9.4 (SAS Institute, Cary, NC). Analyses of survey responses were performed using SURVEY procedures. Weighted prevalence (95% confidence intervals [CI]) of hospitalization either with a primary or secondary *ICD-9-CM* code of JDM were determined. The hospital cost for inpatient care was calculated based on the total charge of the hospitalization and the cost-to-charge ratio estimated by HCUP. All costs were adjusted for inflation to the year 2014 according to the Consumer Price Index from the United States Bureau of Labor Statistics. [14] Summary statistics were generated for length of stay (LOS), inflation-adjusted cost-of-care, including sum, mean and 95% confidence interval (CI) for hospitalizations with a primary, secondary or no diagnosis of JDM.

Three different regression models were constructed. [1] Survey logistic regression models were used to determine the predictors of hospitalization for JDM. The dependent variable was hospitalization with a primary diagnosis of JDM vs. no JDM. Linear regression models with log-transformed [2] cost of care or [3] length of stay (LOS) as the dependent variables were used to determine the predictors of cost of hospitalization and length of stay LOS. Cost of care and LOS were log-transformed because they were not normally distributed. The independent variable was a primary diagnosis of JDM vs. no JDM. Other independent variables included age (0–5, 6–11, 11–17), season of admission (fall, winter, spring, summer), sex (male, female), race/ethnicity (White, Black, Hispanic, Other[Asian, Native American, and other racial/ethnic groups]), health insurance coverage (Medicaid, private, self-pay, no charge/charity, non-Medicaid government administered insurance programs [e.g. KidCare, Children’s Health Insurance Program (CHIP), other federal/state/local government]) number of comorbid chronic conditions (0–1, 2–5, ≥6), hospital location (metropolitan [≥1 urban cluster of population ≥ 50,000] >1million, fringe/metro < 1 million, micropolitan [≥1 urban cluster of population 10,000-49,999], not metropolitan or micropolitan), hospital region (Northeast, Midwest, South and West), and an indicator for calendar year (2002–2003, 2004–2005, 2006–2007, 2008–2009, 2010–2011, 2012). Chronic conditions were defined by HCUP as lasting ≥12 months and meeting one or both of the following: (a) places limitations on self-care, independent living, and social interactions (b) results in the need for ongoing intervention with medical products, services, and special equipment. [15] Chronic condition count was calculated and provided by HCUP. Crude odds ratios (OR), beta-coefficients and 95% CI were estimated. Multivariate regression models were constructed using stepwise selection (alpha = 0.1) from the abovementioned covariates. Adjusted OR, beta coefficients and 95% CI were estimated. All statistical models included discharge trend weights, sample strata that account for hospital’s census region or division, ownership/control, location/teaching and bedsize that were provided by NIS and clustering by individual hospital. Complete case-analysis was performed. A two-sided *P*-value < 0.05 was considered statistically significant.

## Results

### Juvenile dermatomyositis patient and hospital characteristics

**Fig. 1:**
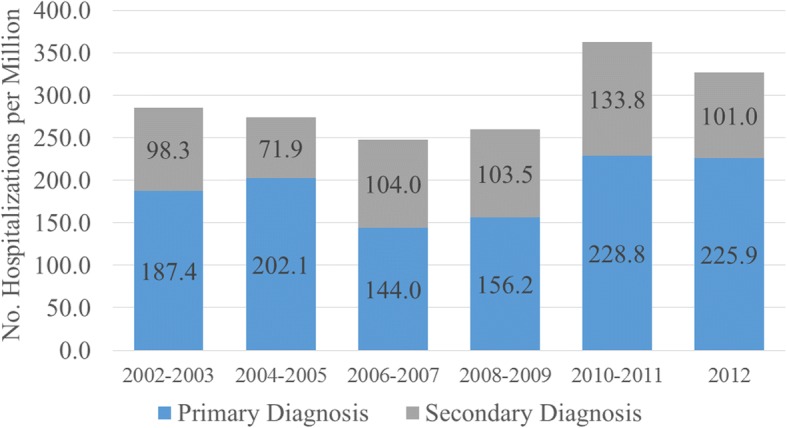
Annual prevalence of hospitalizations for patients with primary/secondary diagnoses of juvenile dermatomyositis (JDM). Survey weighted logistic regression was performed to compare the prevalence of hospitalization for JDM over time. Hospitalization rates for patients with a primary or secondary diagnosis of JDM did not significantly increase after 2003 compared with years 2002–2003 (generalized linear models, *P* < 0.05)

Overall, there were 14,401,668 pediatric discharges captured in the NIS between the years 2002–2012. 4,879,511 pediatric discharges remained after exclusion of live births and other connective tissue diseases. There were 909 and 495 admissions with a primary or secondary diagnosis of JDM (weighted frequencies of 4317 and 2321, respectively). The weighted prevalences of primary and secondary hospitalization for JDM ranged from 144.0–228.8 and 71.9–133.8 per million patients per year (Fig. 1). Hospitalization rates for patients with a primary or secondary diagnosis of JDM did not significantly increase after 2003 compared with years 2002–2003 (generalized linear models, *P* < 0.05; Fig. 1).

Pediatric patients with a primary or secondary diagnosis of JDM were significantly older than those without such a diagnosis (mean [standard deviation] age, 8.77 [0.25] and 10.23 [0.30] vs. 6.19[0.05] years). Hospitalizations with a primary diagnosis of JDM were associated with older patient age compared to hospitalizations without a primary diagnosis of JDM (survey logistic regression; OR [95% CI]) (6–11: 5.79 [4.23–7.93]; 11–17: 2.57 [1.87–3.51]) (Table 1). Patients who were admitted for a primary diagnosis of JDM were more likely to be female (2.17 [1.74–2.71]), receive financial coverage from non-Medicaid government administered insurance programs (2.59 [2.26–2.97]) compared to private insurance, and have multiple chronic conditions (2–5: 1.89 [1.45–2.45]), but were less likely to have Medicaid (0.66 [0.54–0.81]) compared to private insurance. Primary admissions for JDM were less likely to occur in hospitals in nonmetropolitan areas (fringe area or metropolitan area with < 1 million people: 0.65 [0.43–0.99]; micropolitan: 0.30 [0.15–0.57]; not metropolitan or micropolitan: 0.24 [0.13–0.45]) and more likely to occur during the summer (1.44 [1.15–1.80]).

**Table 1:** Associations of hospitalization for JDM in US children

Variable	Primary diagnosis of JDM							
	No		Yes					
	Freq	Row Percent [95% CI]	Freq	Percent [95% CI]	Crude OR [95% CI]	*P*	Adjusted OR [95% CI]	*P*
Age								
0–5	12,470,783	55.00 [54.35–55.64]	1077	25.18 [19.85–30.52]	Reference	–	Reference	–
6–11	3,371,272	14.87 [14.65–15.09]	1686	39.42 [33.59–45.25]	5.79 [4.23–7.93]	<.0001	4.62 [4.18–5.10]	<.0001
11–17	6,832,983	30.13 [29.47–30.80]	1514	35.39 [29.87–40.91]	2.565 [1.872–3.514]	<.0001	1.86 [1.67–2.06]	<.0001
Season								
Fall	5,305,485	25.01 [24.89–25.14]	958	25.29 [21.95–28.64]	1.21 [0.97–1.52]	0.0980	1.18 [1.06–1.33]	0.004
Winter	6,135,926	28.93 [28.69–29.17]	916	24.18 [20.81–27.54]	Reference	–	Reference	–
Spring	5,007,154	23.61 [23.51–23.71]	892	23.53 [20.49–26.57]	1.19 [0.97–1.46]	0.0931	1.13 [1.01–1.27]	0.0349
Summer	4,761,755	22.45 [22.29–22.61]	1023	27.00 [23.73–30.27]	1.44 [1.15–1.80]	0.0015	1.53 [1.37–1.71]	<.0001
Gender								
Female	10,343,988	54.16 [54.03–54.30]	2753	64.74 [59.70–69.70]	2.170 [1.739–2.708]	<.0001	2.22 [2.05–2.42]	<.0001
Male	12,222,939	45.84 [45.70–45.97]	1499	35.26 [30.21–40.30]	Reference	–	Reference	–
Race								
White	9,127,707	51.60 [49.63–53.57]	1712	51.83 [43.21–60.45]	Reference	–	Reference	–
Black	2,970,647	16.79 [15.48–18.11]	529	16.02 [11.32–20.71]	0.949 [0.650–1.388]	0.7889	0.83 [0.73–0.94]	0.0026
Hispanic	3,975,465	24.47 [20.45–24.50]	811	24.56 [17.39–31.72]	1.088 [0.731–1.619]	0.6781	1.01 [0.91–1.13]	0.8044
Other	1,615,416	9.13 [8.24–10.03]	251	7.60 [3.71–11.48]	0.828 [0.462–1.485]	0.5271	0.60 [0.51–0.71]	<.0001
Insurance								
Medicaid	10,669,493	46.42 [45.17–47.66]	1530	35.47 [30.99–39.95]	0.658 [0.538–0.805]	<.0001	0.71 [0.65–0.78]	<.0001
Private insurance	10,435,364	45.40 [44.06–46.74]	2273	52.71 [47.02–58.40]	Reference	–	Reference	–
Self-pay	855,164	3.72 [3.28–4.16]	105	2.44 [0.21–4.66]	0.564 [0.226–1.408]	0.2197	0.57 [0.43–0.76]	<.0001
No charge	43,330	0.19 [0.12–0.26]	9	0.20 [0.00–0.48]	0.903 [0.226–3.606]	0.8850	1.91 [0.97–3.76]	0.0595
Non-Medicaid government program	888,190	3.86 [3.53–4.20]	396	9.18 [5.08–13.29]	2.047 [1.253–3.344]	0.0043	2.59 [2.26–2.97]	<.0001
Number of Chronic Conditions								
0–1	16,472,397	71.53 [70.36–72.70]	2479	57.42 [50.81–64.03]	Reference	–	Reference	–
2 to 5	5,928,336	25.74 [24.77–26.72]	1682	38.96 [33.40–44.52]	1.885 [1.451–2.449]	<.0001	1.41 [1.30–1.54]	<.0001
6+	627,835	2.73 [2.48–2.97]	156	3.62 [1.47–5.76]	1.653 [0.844–3.236]	0.1426	1.24 [1.00–1.52]	0.046
Hospital Location								
Metropolitan >1million	7,153,680	37.36 [33.68–41.04]	19,300	51.32 [40.24–62.41]	Reference	–	Reference	–
Fringe/Metro < 1 million	9,216,267	48.13 [44.80–51.47]	1624	43.19 [32.59–53.79]	0.653 [0.431–0.989]	0.0443	0.70 [0.64–0.77]	<.0001
Micropolitan	1,736,350	9.07 [8.20–9.94]	139	3.69 [1.48–5.91]	0.296 [0.154–0.572]	0.0003	0.27 [0.21–0.35]	<.0001
Not metropolitan or micropolitan	1,041,038	5.44 [4.92–5.95]	67	1.79 [0.73–2.86]	0.240 [0.127–0.454]	<.0001	0.25 [0.18–0.34]	<.0001
Region								
Northeast	4,281,720	18.59 [15.96–21.23]	814	18.87 [7.69–30.04]	Reference	–	Reference	–
Midwest	4,853,593	21.08 [17.99–24.16]	883	20.45 [10.90–29.99]	0.956 [0.463–1.976]	0.9035	0.82 [0.71–0.94]	0.0047
South	8,992,204	39.05 [35.41–42.69]	1450	33.59 [21.57–45.61]	0.848 [0.421–1.708]	0.6441	0.83 [0.74–0.94]	0.0020
West	4,901,051	21.28 [18.32–24.25]	1170	27.10 [15.36–38.84]	1.255 [0.599–2.632]	0.5470	1.23 [1.11–1.36]	<.0001

Missing data was encountered in 74,174 (1.7%) for age, 400,342(10.5%) for season, 96,625 (2.3%) for sex, 1,120,507 (26.2%) for race, 8898, (0.2%) for insurance, 0 (0.0%) for number of chronic conditions, 830,247 (19.7%) for hospital location, and 0 (0.0%) for region

There was no significant differences of missing values for season (*p* = 0.37), race/ethnicity (*p* = 0.76), or hospital location (*p* = 0.14) between hospitalizations with a primary, secondary, or no diagnosis of JDM. 95% *CI* 95% confidence interval; *OR* odds ratio

In multivariate logistic regression models with stepwise selection, non-white race, Medicaid insurance, self-pay, non-metropolitan area, and Midwest and South regions were all associated with lower rates of admission for JDM patients compared to those without a primary diagnosis of JDM, whereas older age, female sex, non-Medicaid government administered insurance coverage, multiple chronic conditions, non-winter seasons and West region were all associated with higher rates of admission for JDM compared to those without a primary diagnosis of JDM (Table 1).

### Reasons for secondary admission

The top 3 primary admission diagnoses for patients with a secondary diagnosis of JDM were: cellulitis of leg (rank, prevalence [95% CI]) (#1, 4.38% [3.46–5.30]), cellulitis of arm (#2, 4.22% [3.31–5.12]), and pneumonia (#3, 3.66% [2.81–4.51]). Meanwhile, the top 3 primary admission diagnoses for inpatients without a diagnosis of JDM were: pneumonia (#1, 5.24% [5.23–5.25]), acute bronchiolitis due to RSV (#2, 3.34% [3.33–3.35], and asthma with exacerbation (#3, 3.04% [3.03–3.05]) (Table 2).

**Table 2:** Top five primary diagnoses of patients admitted with secondary or no diagnosis of JDM

Rank	ICD-9-CM Code	Primary diagnosis	Weighted Frequency	Prevalence [95% CI]
Secondary diagnosis of JDM				
1	6826	Cellulitis of Leg	101.62064	4.38%[3.46–5.30]
2	6823	Cellulitis of Arm	97.86453	4.22%[3.31–5.12]
3	486	Pneumonia	84.98956	3.66%[2.81–4.51]
4	V5789	Rehabilitation procedure NEC	84.56049	3.64%[2.77–4.51]
5	88	Other arthropod-borne disease	52.25267	2.25%[1.58–2.93]
No diagnosis of dermatomyositis				
1	486	Pneumonia	1,206,317	5.24%[5.23–5.25]
2	46,611	Acute Bronchiolitis Due to RSV	768,637	3.34%[3.33–3.35]
3	49,392	Asthma, unspecified, with exacerbation	700,180	3.04%[3.03–3.05]
4	46,619	Acute Bronchiolitis Due to Other Infectious Organism	561,443	2.44%[2.43–2.45]
5	5409	Acute appendicitis w/o mention of peritonitis	550,923	2.39%[2.39–2.40]

ICD-9-CM International Classification of Diseases, Ninth Revision, Clinical Modification; 95% CI 95% confidence interval; RSV respiratory syncytial virus; NEC Not Elsewhere Classifiable

The frequency of diagnosis codes for the most common symptoms in JDM as documented in other studies [16, 17] was also analyzed (Table 3). Compared to non-JDM patients, ten of the most common symptoms (rash, muscle weakness, muscle pain, fever, dysphagia, abdominal pain, arthritis, calcinosis, melena, and anorexia) were more frequent in JDM patients. However, the prevalence of *ICD-9-CM* codes for these symptoms within the dataset was lower than documented prevalence of these symptoms within other published studies [16, 17].

**Table 3:** Primary or Secondary Diagnoses of Most Common Symptoms in Patients with Secondary and No diagnosis of JDM from 2002 to 2012

Rank	*ICD-9-CM* Code^a^	Diagnosis	Weighted Frequency	Row Prevalence [95% CI]
710.3 as Secondary Diagnosis				
1	782.1	Rash	44	1.87 [0.65–3.10]
2	728.87	Muscle Weakness	40	1.72 [0.39–3.05]
3	729.1	Muscle Pain	30	1.30 [0.33–2.28]
4	780.6	Fever	96	4.14 [2.26–6.01]
5	787.20	Dysphagia	45	1.94 [0.43–3.46]
6	784.42	Dysphonia	0	–
7	789.00	Abdominal Pain	23	0.97 [0.11–1.83]
8	CCS 204	Arthritis NOS	80	3.44 [1.86–5.02]
9	709.3	Calcinosis	27	1.14 [0.06–2.23]
10	578.1	Melena	8	0.36 [0.00–0.86]
11	783.0	Anorexia	10	0.43 [0.00–1.04]
No Diagnosis of 710.3				
1	782.1	Rash	166,932	0.73 [0.68–0.77]
2	728.87	Muscle Weakness	8152	0.04 [0.03–0.04]
3	729.1	Muscle Pain	31,879	0.14 [0.13–0.15]
4	780.6	Fever	621,463	2.70 [2.53–2.87]
5	787.20	Dysphagia	64,240	0.28 [0.23–0.32]
6	784.42	Dysphonia	1836	0.01 [0.00–0.01]
7	789.00	Abdominal Pain	159,317	0.69 [0.65–0.73]
8	CCS 204	Arthritis NOS	136,958	0.59 [0.56–0.63]
9	709.3	Calcinosis	1025	0.0044 [0.0036–0.0053]
10	578.1	Melena	59,509	0.26 [0.24–0.28]
11	783.0	Anorexia	67,501	0.29 [0.25–0.33]

^a^Excluded repeat diagnosis of JDM

ICD9-CM International Classification of Diseases, Ninth Revision, Clinical Modification; 95% CI 95% confidence interval; NOS Not Otherwise Specified

### Length of stay

**Fig. 2:**
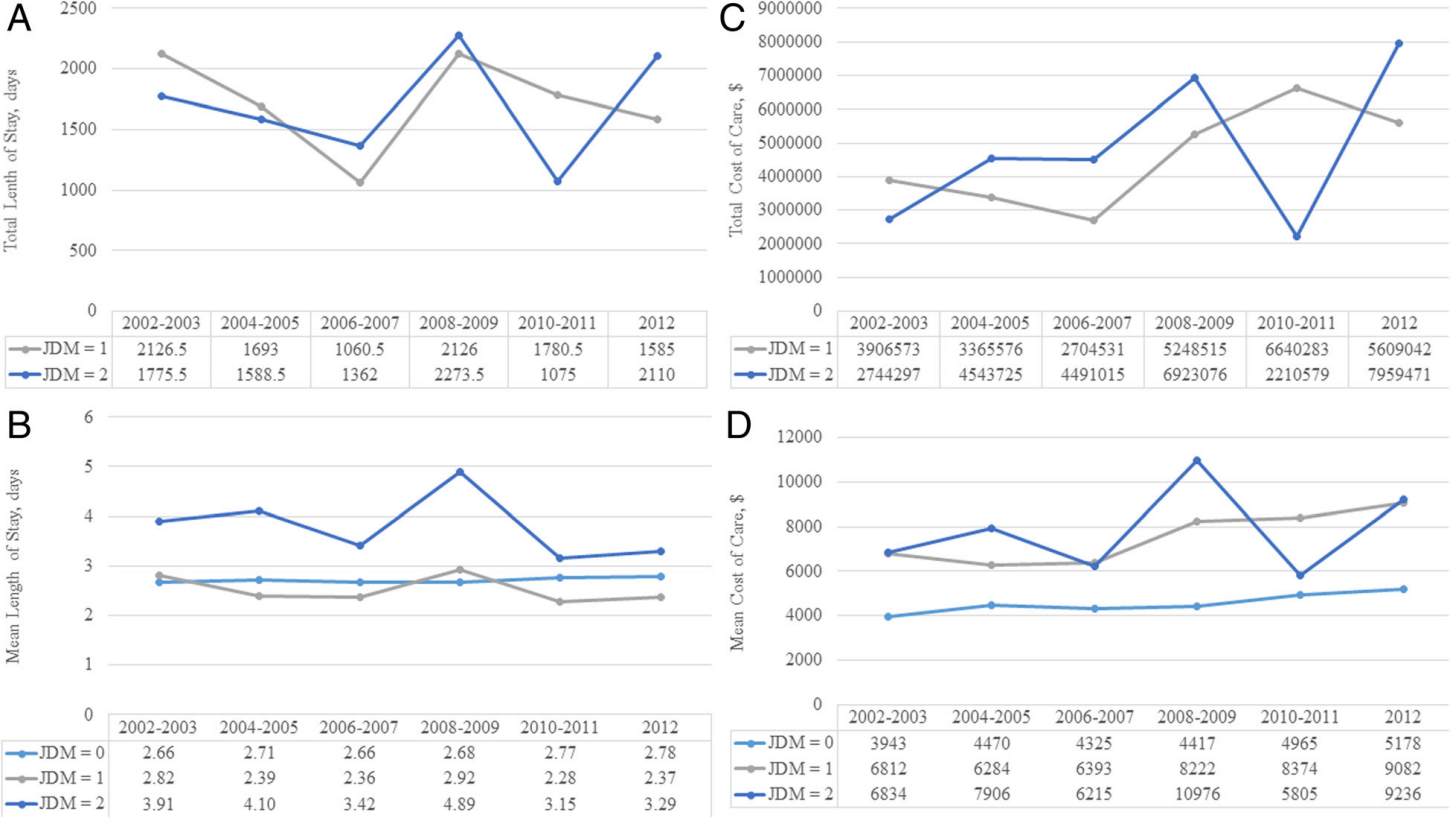
Length of stay and costs of hospitalization with primary/secondary diagnoses for juvenile dermatomyositis (JDM). (**a**) Total and (**b**) geometric mean length of hospital stay and (**c**) total and (**d**) geometric mean inflation-adjusted cost of inpatient care are presented for 2002–2003, 2004–2005, 2006–2007, 2008–2009, 2010–2011 and 2012. *jdm* 0: no JDM. *jdm* 1: primary dx of JDM. *jdm* 2: secondary dx of JDM

Patients with JDM spent a weighted total of 19,159 days and 18,218 days in the hospital for their JDM or other reasons, respectively. LOS in the hospital was 39% longer for hospitalizations with a secondary diagnosis (geometric mean [95% CI]: 3.75 [3.27–4.30] days) (*p* < 0.0001) compared with hospitalizations without a diagnosis of JDM while hospitalizations were not prolonged for a primary diagnosis (2.50 [2.27–2.76]) when compared with hospitalizations without a diagnosis of JDM (2.70 [2.66–2.75]). This pattern of prolonged LOS for hospitalizations with a secondary diagnosis of JDM was consistent across all years (Fig. 2).

In multivariate weighted linear regression models of log-transformed LOS, increased LOS in patients with a primary diagnosis of JDM was associated with race/ethnicity (beta coefficient [95% CI]) (Hispanic: 0.28 [0.14–0.41]; Other: 0.59 [0.31–0.86]), type of insurance (Medicaid: 0.16 [0.01–0.31]), multiple chronic conditions (2–5: 0.41 [0.29–0.52]; 6+: 0.94 [0.63–1.26]), micropolitan location (0.59 [0.27–0.90]), and South region (0.34 [0.12–0.56]) (Table 4). Note that since LOS was log transformed, coefficients from regression models of log-transformed LOS are not the same scale as raw LOS.

**Table 4:** Predictors of Length of Stay and Cost of Care for Hospitalizations with a Primary Diagnosis of JDM-

	Length of Stay			Cost of Care		
	LSM	Adj Beta [95% CI]^a^	*p*-value	LSM	Adj Beta [95% CI]*	*p*-value
Age						
0–5	1.72	0 [ref]	–	9.47	0 [ref]	–
6–10	1.51	−0.22[− 0.34--0.09]	0.001	9.43	− 0.03[− 0.24–0.17]	0.7459
11–17	1.69	− 0.04[− 0.19–0.12]	0.6417	9.64	0.18[− 0.07–0.42]	0.1536
Season						
Fall	1.62	−0.05[− 0.16–0.06]	0.3442	9.57	0.00[− 0.24–0.23]	0.9674
Winter	1.67	0 [ref]	–	9.57	0 [ref]	–
Spring	1.6	−0.07[− 0.15–0.01]	0.0853	9.45	− 0.13[− 0.35–0.09]	0.2514
Summer	1.66	− 0.01[− 0.11–0.08]	0.7552	9.46	− 0.11[− 0.33–0.10]	0.3131
Gender						
Female	1.64	0.01[− 0.09–0.11]	0.8992	9.54	0.05[− 0.13–0.23]	0.5914
Male	1.63	0 [ref]	–	9.49	0 [ref]	–
Race						
White	1.38	0 [ref]	–	9.32	0 [ref]	–
Black	1.55	0.17[− 0.12–0.46]	0.2395	9.44	0.12[− 0.21–0.45]	0.4806
Hispanic	1.66	0.28 [0.14–0.41]	<.0001	9.62	0.30 [0.05–0.55]	0.0168
Other	1.96	0.59 [0.31–0.86]	<.0001	9.68	0.36[−0.07–0.79]	0.0993
Insurance						
Medicaid	1.62	0.16 [0.01–0.31]	0.0386	9.32	0.02[−0.21–0.25]	0.8646
Private insurance	1.46	0 [ref]	–	9.3	0 [ref]	–
Self-pay	1.7	0.24[−0.07–0.55]	0.1316	9.52	0.22[−0.67–1.10]	0.6276
No charge	2.11	0.65[−0.06–1.37]	0.0739	10.48	1.18[−0.56–2.93]	0.1833
Non-Medicaid government program	1.3	−0.16[− 0.29--0.04]	0.0085	8.95	−0.34[− 0.63--0.06]	0.0196
Number of Chronic Conditions						
0–1	1.19	0 [ref]	–	9.06	0 [ref]	–
2 to 5	1.59	0.41 [0.29–0.52]	<.0001	9.42	0.36 [0.17–0.55]	0.0002
6+	2.13	0.94 [0.63–1.26]	<.0001	10.07	1.01 [0.39–1.63]	0.0014
Hospital Location						
Metropolitan >1million	1.46	0 [ref]	–	9.52	0 [ref]	–
Fringe/Metro < 1 million	1.41	−0.04[−0.14–0.05]	0.3704	9.35	−0.17[− 0.36–0.02]	0.0835
Micropolitan	2.04	0.59 [0.27–0.90]	0.0003	9.87	0.35[−0.20–0.91]	0.2128
Not metropolitan or micropolitan	1.64	0.19[−0.69–1.07]	0.6753	9.31	−0.20[− 0.98–0.58]	0.6082
Region						
Northeast	1.62	0 [ref]	–	9.35	0 [ref]	–
Midwest	1.51	−0.10[−0.29–0.09]	0.285	9.63	0.28[−0.04–0.59]	0.0875
South	1.95	0.34 [0.12–0.56]	0.0024	9.63	0.28 [0.00–0.56]	0.0529
West	1.47	−0.15[−0.35–0.05]	0.1342	9.44	0.09[−0.17–0.35]	0.5113
Year						
2002–2003	1.79	0 [ref]	–	9.55	0 [ref]	–
2004–2005	1.64	−0.15[−0.31–0.02]	0.0785	9.44	−0.11[− 0.44–0.23]	0.5253
2006–2007	1.53	−0.26[− 0.47--0.05]	0.0146	9.34	− 0.21[− 0.57–0.15]	0.2445
2008–2009	1.73	−0.06[− 0.24–0.11]	0.4759	9.61	0.06[− 0.29–0.40]	0.7548
2010–2011	1.51	− 0.18[− 0.49–0.14]	0.2666	9.39	−0.16[− 0.52–0.20]	0.3796
2012	1.61	−0.28[− 0.43--0.13]	0.0003	9.75	0.20[− 0.14–0.54]	0.2469

Missing data was encountered in 74,174 (1.7%) for age, 400,342(10.5%) for season, 96,625 (2.3%) for sex, 1,120,507 (26.2%) for race, 8898, (0.2%) for insurance, 0 (0.0%) for number of chronic conditions, 830,247 (19.7%) for hospital location, 0 (0.0%) for region, and 0 (0.0%) for year

There were no significant differences of missing values for season (*p* = 0.37), race/ethnicity (*p* = 0.76), or hospital location (*p* = 0.14) between hospitalizations with a primary, secondary, or no diagnosis of JDM. LSM least squares mean; 95% *CI* 95% confidence interval

^a^Coefficients from regression models of log-transformed LOS or cost of care should be interpreted with caution as the transformed variables are not the same scale as the raw variables

### Cost of care

The weighted total inflation-adjusted cost-of-care for patients with a primary and secondary inpatient diagnosis of JDM was $49,339,995 and $49,784,853 respectively. The actual total cost is likely higher as 106 patients had a missing value for charge and cost. The inflation-adjusted cost of care for hospitalization was 64% higher for hospitalizations with a primary diagnosis (geometric mean [95% CI]: $7350 [$6228–$8674] and 64% higher for a secondary diagnosis ($7352 [$6331–$8537]) of JDM than those with no diagnosis of JDM (4479 [$4295–$4671] (*p* < 0.0001 for both). This pattern of higher costs for hospitalizations for JDM was consistent for every year within the cohort.

In multivariate linear regression models of log-transformed cost of care, increased cost-of-care in patients with a primary diagnosis of JDM was associated with race/ethnicity (beta coefficient [95% CI] Hispanic: 0.30 [0.05–0.55]) and multiple chronic conditions (2–5: 0.36 [0.17–0.55]; 6+: 1.01 [0.39–1.63]).

## Discussion

The present study finds that JDM incurs a significant inpatient burden, with primary diagnoses having a higher cost of care (approximately $3000 more per hospitalization) and secondary diagnoses having longer hospitalizations and cost of care (also approximately $3000 more per hospitalization) compared to no diagnosis of JDM. The higher cost of care is likely related to additional workup required for diagnosis, disease comorbidity and treatment regimen required for the disease. Previous studies have suggested that infection may play a role in both the development [18] and recurrence [19, 20] of JDM episodes. In our study, we found increased rates of infection i.e. cellulitis among JDM patients. Multiple mechanisms may make individuals with JDM more prone to infection with pathogens such as staphylococcus. Skin breakdown, seen in JDM patients with calcinosis and cutaneous ulceration, may serve as a nidus for infection. In addition, underlying immune dysregulation such as granulocyte chemotactic defects may contribute to infection risk. [21] The long-term use of immunosuppressants (e.g. corticosteroids, methotrexate) likely also contributes to infection risk. A novel finding that ‘other arthropod-borne diseases’ represented the 5th most common primary diagnosis among patients with a secondary diagnosis of JDM (Table 2) deserves further study to determine the strength of this association, whether the code refers specifically to arthropod-borne diseases per se versus chief complaints (e.g. cutaneous eruptions, other infections) for which arthropod-borne diseases are on the differential diagnosis, or an outlier due to readmission of a few inpatients with JDM who happened to have had arthropod-borne diseases. JDM patients within our study were also shown to have higher rates of comorbid chronic conditions. Emerging literature supports the finding that JDM patients are often medically complex. For example, we recently found in a separate study that inpatients with JDM had increased odds of multiple cardiovascular and cerebrovascular comorbidities. [22] It is likely that sequelae of JDM itself, adverse effects of treatment (e.g. infections, side effects of steroids), and concomitant autoimmunity contribute to higher chronic condition counts in JDM, with further study needed to better clarify the exact comorbidities in this population. In addition, while current treatment regimens favoring rapid and aggressive management, such as those involving high-dose intravenous pulse methylprednisone therapy, improve outcomes and prognosis of the disease [23], they may also contribute to inpatient costs. Unfortunately, we did not have access to specific diagnostic tests and medications used during hospitalizations, as these were not recorded within the NIS. As a result, future studies are needed to determine specific contributors to inpatient costs. Notably, annual prevalence for both primary and secondary diagnoses have not increased over time as opposed to trends seen in adult dermatomyositis. [6] Future studies are needed to assess whether disease pathophysiology or management may explain the differences in these trends.

There were significant age, sex, seasonal, financial, and regional differences in hospitalization for JDM. In particular, higher rates of hospitalization were found in JDM patients who were older, female, received non-Medicaid government administered insurance coverage (e.g. KidCare, CHIP, other federal/state/local government programs), hospitalized in metropolitan areas, and hospitalized in non-winter seasons when compared to the control group. Hospitalization rates were highest for the 6–11 year old age group, which corresponds to the average age of onset (approximately 7 years) found in other studies. [18, 24] Higher rates of hospitalization in females are likely related to females having higher overall disease prevalence. [2, 18] Hospitalizations for JDM were more likely to occur in non-winter seasons, which stand in stark contrast to the higher frequency of winter admissions seen in control patients. This finding reinforces that ultraviolet light and other environmental exposures may play a role in disease pathogenesis and exacerbation, which corroborates the view that JDM can be a photosensitive condition [25–27] and that JDM patients should be advised to routinely use sunscreen. [28]

Financial coverage and regional differences in hospitalizations may point to differences in access to care for different patient populations. Notably, patients were more likely to be hospitalized if they were covered under government programs, excluding Medicaid. These alternative programs often represent efforts at the state/local level to provide assistance for children of lower income families. As a result, increased risk of hospitalization for patients with these alternative forms of coverage may represent the decreased access to care that lower income families face. Paradoxically, hospitalizations rates were lower among JDM patients with Medicaid coverage. As a result, further research is needed to evaluate how differences in group characteristics and social determinants of health between those covered under different government programs may contribute to differences in outcomes as seen in this study. Additionally, the majority of hospitalizations occurred in metropolitan locations. This speaks to multiple potential issues, including access to outpatient care in urban settings and lack of pediatric rheumatologists in more rural settings requiring treatment at large referral centers in urban areas, both of which may be attributable to a limited supply of pediatric rheumatologists [29] and pediatric subspecialists in general. [30] This is further reinforced by the fact that hospitalizations for JDM in micropolitan locations resulted in an 80% longer LOS compared to metropolitan locations. Further research is needed to evaluate how distribution and access to the pediatric rheumatology workforce affects patient outcomes.

Interestingly, race/ethnicity did not play a major role in increased hospitalizations, contrasting trends seen across other diseases, including adult dermatomyositis [6], pemphigus [12], and psoriasis [31] where racial/ethnic differences did affect rates of hospitalization. However, race was an important contributor to inpatient burden in terms of both length of stay and cost, with Hispanic patients having an estimated 32% longer hospitalization and 35% higher cost of care and other non-white racial groups (excluding Black and Hispanic patients) having 80% longer hospitalizations. These differences in length of stay and costs of care may point to differences in disease manifestations or severity among different races/ethnicities. Indeed, previous studies have found differences in human leukocyte antigen (HLA) frequencies among different ethnic groups, with the HLA-DQA1*0501 allele having a high frequency in Hispanic JDM patients. [32] In addition, studies have suggested that differences between patient HLA subtype and autoantibody associations may help predict disease subtype and severity. [33] Alternatively, access to care may be contributing to racial/ethnic differences. However, one study, which found that minority race and lower family income were associated with worse outcomes—as measured by Childhood Health Assessment Questionnaire, patient global scores, and health-related quality of life scores—did not find associations between race and proxy measures of access to care e.g. time to diagnosis, disease duration, and treatment. [34] In our own study, we did not find that Black patients experienced a difference in hospitalization, length of stay, or cost of care, although we did find that these outcomes differed among other minority groups. Further research examining the relationship of race/ethnicity to outcomes in JDM are warranted.

Strengths of this study include an analysis of a nationally representative sample of all payer data over a period of 11 years with over 14 million pediatric records. JDM patients were identified using the previously validated *ICD-9-CM* codes for dermatomyositis in the inpatient setting. [13] Limitations of this study include the inability to distinguish between different degrees of disease severity or specific clinical features. This limited our ability to examine how differences between individual hospitals and patient characteristics might contribute to LOS and cost of care. Due to the structure of the NIS dataset, it was not possible to determine how many of the hospitalizations were due to readmissions or transfers between hospitals. In addition, cost analysis did not include costs of physician services, out-of-pocket expenses or outpatient costs. Thus the total economic burden of JDM is likely much higher. In addition, while common symptoms for JDM were higher among the JDM cohort, little additional insight could be gleaned due to lack of symptom coding within the database, as prevalence of these codes was much lower than documented prevalence of these symptoms. [16, 17] There was a large frequency of missing data for season, race/ethnicity, and hospital location in NIS. However, there were no significant differences of missing values between hospitalizations with a primary, secondary or no diagnosis of dermatomyositis. Finally, geographic variation was considered by four Health Resources and Services Administration regions. Controlling for region did not attenuate the observed seasonal, racial/ethnic, or hospital location differences. However, future studies using more granular distinctions of geographic location would be useful to further validate these seasonal, racial, and hospital location differences.

## Conclusions

The findings of this study indicate that the inpatient burden of JDM is extensive. Cost was higher for patients with primary JDM while both length of stay and cost were higher for secondary dermatomyositis versus those without. Older age, female sex, non-winter season, non-Medicaid administered government insurance coverage, and metropolitan area were associated with higher rates of hospitalization for JDM while race/ethnicity was more influential in resulting in increased LOS and cost of care, particularly in Hispanic populations. Future research is needed to identify how factors such as access to care may relate to the financial, regional, and racial differences found within this study.

## Abbreviations

AHRQ: Agency for Healthcare Research and Quality
CI: Confidence Intervals
HCUP: Healthcare Cost and Utilization Project
HLA: Human Leukocyte Antigen
ICD-9-CM: International Classification of Diseases, Ninth Revision, Clinical Modification
JDM: Juvenile dermatomyositis
LOS: Length of Stay
NEC: Not Elsewhere Classifiable
NIS: Nationwide Inpatient Sample
NOS: Not Otherwise Specified
OR: Odds Ratio
RSV: Respiratory Syncytial Virus
